# Gender differences in the impact of 3-year status changes of metabolic syndrome and its components on incident type 2 diabetes mellitus: a decade of follow-up in the Tehran Lipid and Glucose Study

**DOI:** 10.3389/fendo.2023.1164771

**Published:** 2023-05-25

**Authors:** Farzad Hadaegh, Amir Abdi, Karim Kohansal, Parto Hadaegh, Fereidoun Azizi, Maryam Tohidi

**Affiliations:** ^1^ Prevention of Metabolic Disorders Research Center, Research Institute for Endocrine Sciences, Shahid Beheshti University of Medical Sciences, Tehran, Iran; ^2^ Student Research Committee, School of Medicine, Tehran Medical Sciences, Islamic Azad University, Tehran, Iran; ^3^ Endocrine Research Center, Research Institute for Endocrine Sciences, Shahid Beheshti University of Medical Sciences, Tehran, Iran

**Keywords:** metabolic syndrome, type 2 diabetes mellitus, gender, change, risk

## Abstract

**Background:**

The aim of this study was to examine the gender differences in the association between status changes of metabolic syndrome (MetS) and its components, using Joint Interim Statement (JIS) criteria, with the risk of type 2 diabetes mellitus (T2DM) among an urban population.

**Methods:**

The study included 4,463 Iranian adult participants (2,549 women) aged ≥20 years. Based on status changes of MetS and its components during 3 years, subjects were categorized into four groups: MetS-free (reference), MetS-developed, MetS-recovery, and MetS-stable. A similar categorization was applied to MetS components. Multivariable Cox regression models were used for estimating hazard ratios (HRs) and women-to-men ratios of HRs (RHRs).

**Results:**

During a median follow-up of 9.3 years, 625 T2DM events (351 women) occurred. Compared with the reference, the HRs of the MetS-developed, -recovery, and -stable groups among men for incident T2DM were 2.90, 2.60, and 4.92; the corresponding values for women were 2.73, 2.88, and 5.21, respectively (all *p*-values < 0.01), without significant gender difference in these relationships. In both genders, the fasting plasma glucose (FPG) component, regardless of the change in status, was strongly and significantly associated with incident T2DM with HRs ranging from 2.49 to 9.42; a similar association was also found for high waist circumference (WC)-recovery and -stable groups, with HRs ranging from 1.58 to 2.85 (*p*-values ≤ 0.05). Regarding gender differences, the development and persistence of high blood pressure (BP) status exposed men to greater T2DM risk than women with women-to-men RHRs of 0.43 (0.26–0.72) and 0.58 (0.39–0.86), respectively. Moreover, stable low levels of high-density lipoprotein cholesterol (HDL-C) and high triglyceride (TG) levels conferred higher T2DM risk in women than in men, with women-to-men RHRs of 1.67 (0.98–2.86) and 1.44 (0.98–2.14), respectively (both *p*-values = 0.06).

**Conclusion:**

Among Tehranian adults, in both genders, all status changes of MetS, even those recovered from MetS, have a higher risk of T2DM compared to those who never had MetS. Also, all statuses of high FPG, in addition to recovered and stable high WC, were strongly associated with T2DM risk. Specifically, men with stable or developed high BP and women with stable dyslipidemic status were at differentially increased risk of incident T2DM.

## 1 Introduction

Metabolic syndrome (MetS) is a cluster of impaired metabolic factors, including elevated fasting glucose, central obesity, high blood pressure (BP), and atherogenic dyslipidemia [i.e., raised triglycerides (TG) and reduced high-density lipoprotein cholesterol (HDL-C)], as first described by Reaven (1). Insulin resistance plays a pivotal role in the pathophysiological mechanisms of MetS; in fact, MetS is considered a surrogate of insulin resistance (IR) (1, 2). MetS has a well-established association with increased risk for type 2 diabetes mellitus (T2DM), cardiovascular diseases (CVD), and mortality (3–8).

A high burden of MetS and T2DM has been reported in studies conducted in the Middle East and North Africa (MENA) region (9–13). In this respect, previous studies in Iran have demonstrated that 1 out of every 3 Iranian adults aged 30 and above has MetS (14). Moreover, approximately 5% and 1% of Iranian adults aged 20 years and above develop MetS and T2DM annually (15, 16), respectively.

It is of note that previous attempts to establish the association of MetS and its outcomes were mainly through a “snapshot” approach. Although numerous studies demonstrated the association of MetS at baseline and the development of T2DM, data on status changes of MetS over a period of time and its impact on the incidence of T2DM are scarce and limited to a few studies conducted in East Asia (17–20). Furthermore, only one of these studies assessed the impact of status alteration of each MetS component and incident T2DM (18).

To the best of our knowledge, gender differences regarding the changes in MetS status and T2DM incidence have not been explored yet. Hence, in the present study, we aimed to use the data from the cohort of the Tehran Lipid and Glucose Study (TLGS) in order to examine the impact of 3-year status changes of MetS and its components as defined by Joint Interim Statement (JIS) criteria (21) on incident T2DM and to compare this impact in women versus men for incident T2DM among an urban population of Tehran during a near-decade of follow-up.

## 2 Materials and methods

### 2.1 Study population

**Figure 1:**
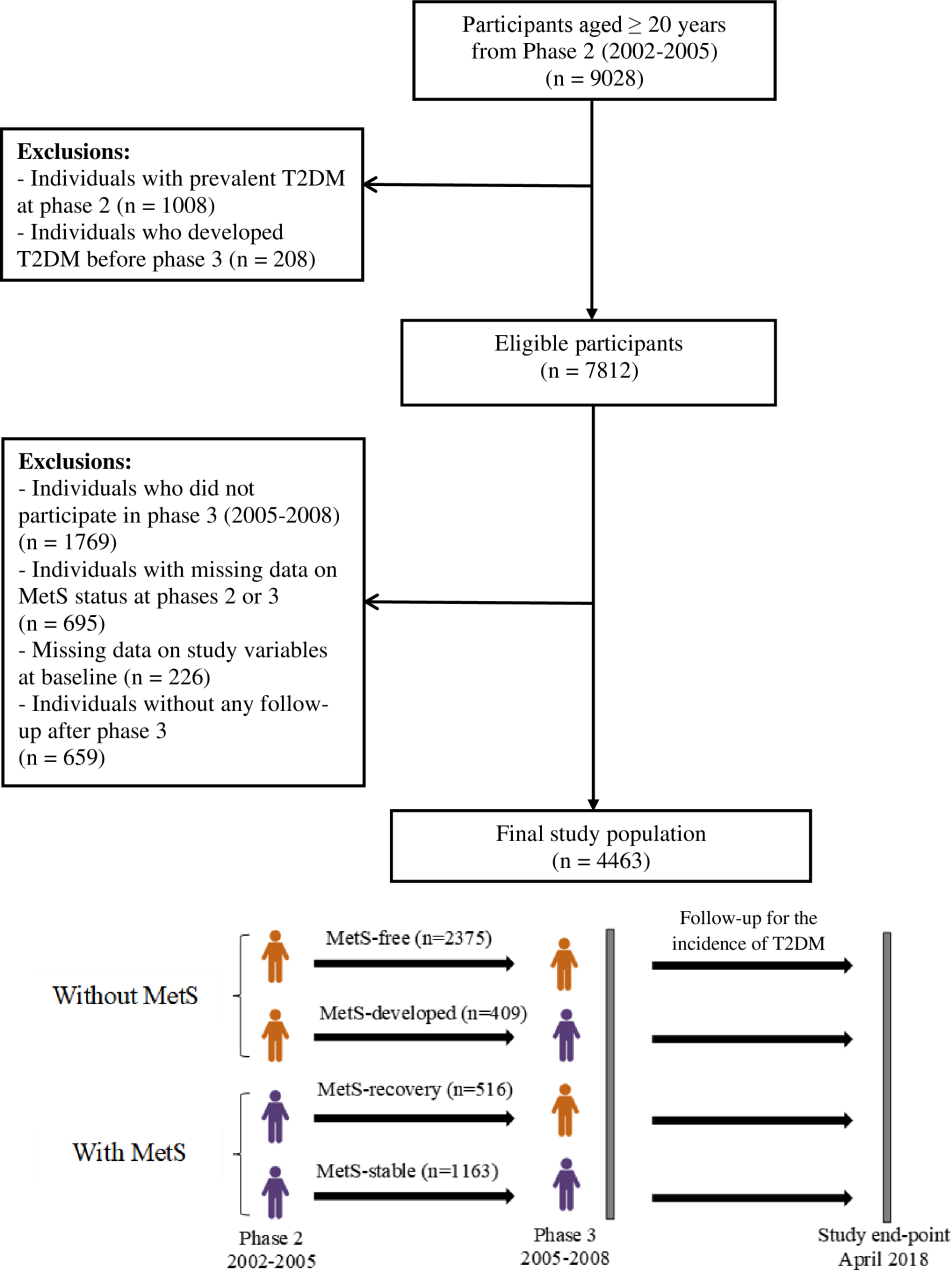
Flow diagram of the study participants. T2DM, type 2 diabetes mellitus; MetS, metabolic syndrome.

TLGS is a population-based prospective cohort study with the primary aim of assessing the prevalence and incidence of non-communicable diseases (NCDs) and related risk factors. Furthermore, another objective of the TLGS is to prevent NCD by implementing a healthy lifestyle through an educational intervention on a sub-population of the cohort. This study was performed on a representative sample of an urban population of Tehran (13th district of Tehran). TLGS recruitment was done during two phases: phase 1 (1999-2002), a total of 15,005 individuals, and phase 2 (2002 to 2005), with 3,550 additional recruitments aged ≥ 3 years enrolled in the study using the multistage cluster random sampling method. After that, the study participants were followed and re-examined triennially in phase 3 (2005–2008), phase 4 (2009–2011), phase 5 (2012–2015), and phase 6 (2015–2018). A more detailed description of the TLGS design and methodology has been reported elsewhere (22, 23). The current study was conducted in the framework of the TLGS on 9,028 subjects aged ≥20 years who participated in phase 2. Those with prevalent T2DM at phase 2 and phase 3 (index year) (*n* = 1,216) were excluded, leaving *n* = 7,812 eligible participants. Other exclusions include those who did not participate in phase 3 (*n* = 1,769), those with missing data on MetS and other covariates (*n* = 921, considering overlap features between numbers), and those without any follow-up after the index year (*n* = 659). The remaining *n* = 4,463 (2,549 women) were followed till April 2018 (**Figure 1**).

This study is approved by the Institutional Review Board of the Research Institute for Endocrine Sciences (RIES), Shahid Beheshti University of Medical Sciences, Tehran, Iran, and each participant provided written informed consent.

### 2.2 Clinical and laboratory measurements

In the TLGS, a questionnaire including information on demographics, marital status, family history of T2DM, education, smoking habits, and medications is completed by a trained interviewer. The Modifiable Activity Questionnaire (MAQ) has been used to assess physical activity levels from phase 2, which measures all three types of activity, including leisure time, job, and household activities in the past year (24).

For the procedure of recording anthropometric measures, the subjects were lightly clothed and without shoes. A digital scale (Seca 707, Seca Corp; range 0.1–150 kg, sensitivity 0.1 kg) was used for weight measurement. Height was measured in a standing position, with shoulders placed in normal alignment using a tape meter. Body mass index (BMI) was calculated as weight (kg) divided by the square of height (m^2^). Waist circumference (WC) was measured at the umbilical level by an upstretched tape meter. For blood pressure measurement, the subjects rested for 15 min; systolic and diastolic blood pressures (SBP and DBP, respectively) were measured twice on the right arm using a standardized mercury sphygmomanometer calibrated by the Iranian Institute of Standards and Industrial Researches. Finally, SBP and DBP were recorded as means of these measurements.

The participants had 12–14 h of overnight fasting before collecting a venous blood sample for measurements of fasting plasma glucose (FPG) and lipids. For all participants not on glucose-lowering medications, an oral glucose tolerance test was performed by taking 82.5 g of glucose monohydrate (equivalent to 75 g of anhydrous glucose); 2 h later, another blood sample was taken to assess 2-h post-challenge glucose. An enzymatic colorimetric method with glucose oxidase was used to measure plasma glucose. TG level was assayed by an enzymatic colorimetric method using lipoprotein lipase and glycerol phosphate oxidase.

After the precipitation of the apolipoprotein-B-containing lipoproteins by phosphotungstic acid, HDL-C was assayed using the enzymatic colorimetric method with cholesterol esterase and cholesterol oxidase. All analyses were done in the TLGS research laboratory on the same day as blood sampling using commercial kits (Pars Azmoon Inc., Tehran, Iran) and a Selectra 2 auto-analyzer (Vital Scientific, Spankeren, The Netherlands). Assayed serum controls in two different concentrations (TruLab N and TruLab P; Pars Azmoon Inc.) were used to monitor the accuracy of measurements. The intra- and inter-assay coefficients of variation (CVs) were both less than 2.3% for glucose, and regarding TG and HDL-C, both intra- and inter-assay CVs were less than 2.1% and 3.0%, respectively.

### 2.3 Definitions

#### 2.3.1 Main exposure: MetS and its components

**Figure 2:**
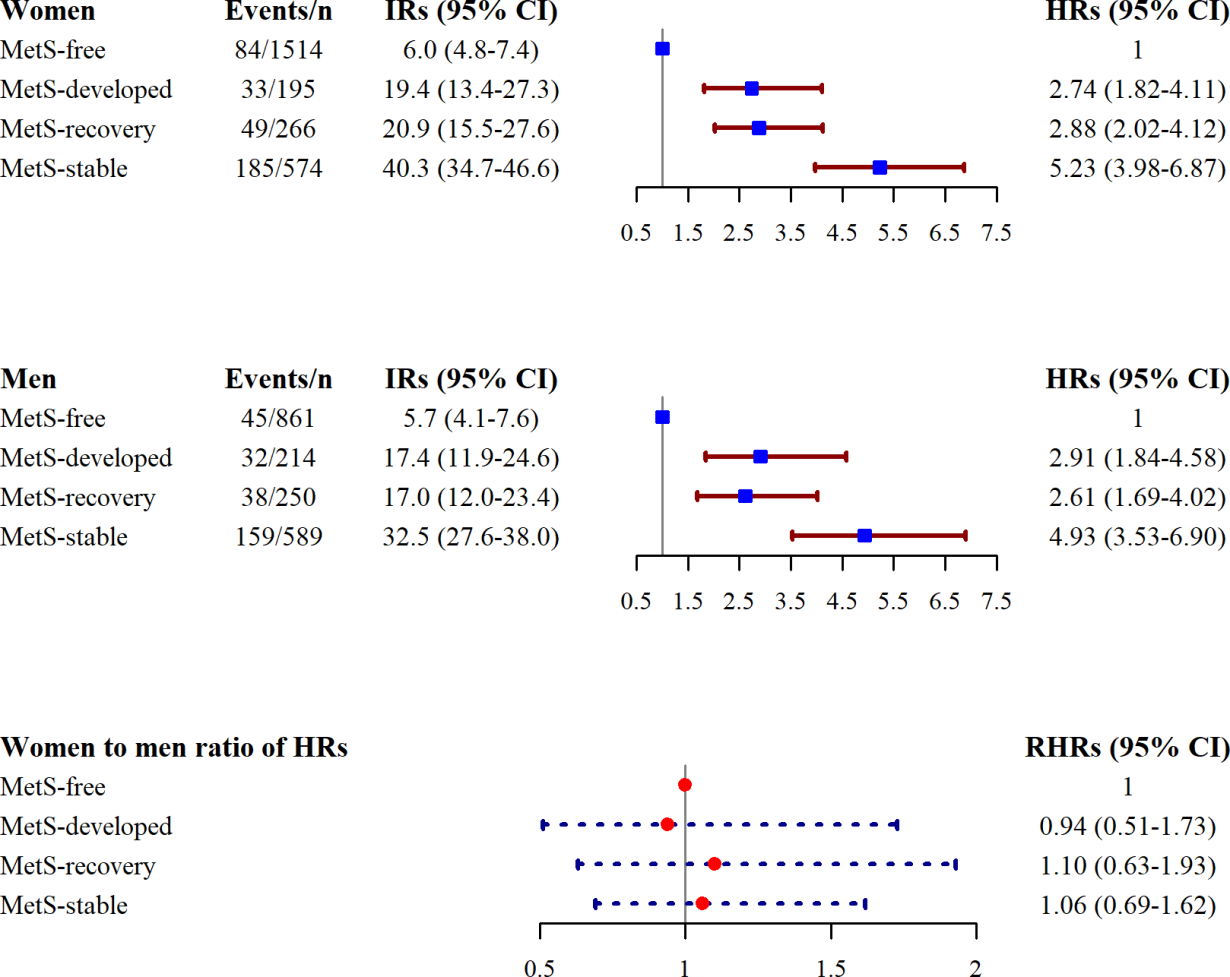
Association of MetS status change with the risk of T2DM, Tehran Lipid and Glucose Study. HR, hazard ratio; RHR, ratio of hazard ratios; IR, incidence rate; *n*, total number of population; MetS, metabolic syndrome; T2DM, type 2 diabetes mellitus. HR was estimated using the Cox regression model adjusted for age, body mass index, smoking status, physical activity, education, marital status, being in the intervention group, and family history of T2DM.

MetS was defined according to the Joint Interim Statement of the International Diabetes Federation Task Force on Epidemiology and Prevention (21). The presence of at least three of the following criteria was defined as MetS: (a) elevated WC (≥90 cm for both genders to identify the Iranian population at risk of CVD risk factors requiring lifestyle change) (25), (b) elevated BP (≥130/85 mmHg or treatment with anti-hypertensive drugs), (c) elevated FPG (≥5.6 mmol/L) or use of glucose-lowering medications, (d) elevated TG (≥1.7 mmol/L) or using lipid-lowering drugs, and (e) low HDL-C (<1.03 mmol/L for men and <1.29 mmol/L for women). We split the study participants into four groups according to the MetS status change (**Figure 2**): (a) subjects without MetS in both phases 2 and 3 (MetS-free as reference); (b) those without MetS at phase 2 and developed MetS at phase 3 (MetS-developed); (c) those with MetS at phase 2 but the absence of MetS at phase 3 (MetS-recovery); and (d) those who had MetS in both phase 2 and phase 3 (MetS-stable). A similar approach was applied for defining different categories for each MetS component, given those without each component in both phases 2 and 3 as the reference.

#### 2.3.2 Outcome and other covariates

T2DM was defined as having at least one of the following criteria: fasting glucose ≥7 mmol/L, 2-h post-challenge glucose ≥11.1 mmol/L, or taking glucose-lowering medication. Individuals with missing data on 2-h post-challenge glucose who simultaneously had fasting glucose < 5.05 mmol/L were considered T2DM-free (26). Diagnosis of T2DM in at least one parent or sibling of the participants was considered a positive family history of T2DM (FH-T2DM).

Regarding the smoking status, three categories were defined: (a) current smoker: a person who smokes cigarettes or other smoking implements daily or occasionally, (b) past-smoker: a person who quit smoking for at least 1 year prior to study entry, (c) never smoker (reference group): a person who had never smoked. Individuals were divided into three categories according to their self-reported education status: less than 6 years (reference group), 6–12 years, and more than 12 years of schooling. Marital status was classified as single (reference group), married, and widowed/divorced. Individuals who scored ≥600 metabolic equivalent task (MET) minutes/week were considered physically active (27).

### 2.4 Statistical analysis

Continuous and categorical variables were presented as mean [standard deviation (SD)], and number (percentage), respectively. For continuous variables with skewed distribution (FPG and TG), the median [interquartile range (IQR)] was shown. We compared baseline characteristics between four groups in both genders based on changes in MetS status. Moreover, we compared baseline characteristics between respondents and non-respondents (including those who were not examined in the index year or not followed after this time, as well as those with missing data). Comparisons were performed using one-way analysis of variance (ANOVA) for continuous variables and the Chi-squared test for categorical variables. Continuous variables with skewed distribution were compared using the Kruskal–Wallis test. A crude incidence rate of T2DM and 95% confidence interval (CI) per 1,000 person-years were calculated for MetS and each component. The multivariable Cox proportional hazards regression models was applied to calculate the hazard ratio (HRs) and 95% CIs for the association between changes in MetS and its components and the risk of incident T2DM, given free states as references.

Model 1 was adjusted for age. Model 2 was further adjusted for smoking status, physical activity level, education, marital status, family history of T2DM, BMI, being in the intervention group, and other components of MetS. To estimate the women-to-men hazard ratios (RHRs), we included an interaction term of each exposure variable (baseline MetS, baseline MetS components, change in MetS, and changes in MetS components) with sex in multivariable Cox models. All analyses were performed using R software (Version 4.1.2), and two-sided *p* < 0.05 was considered statistically significant.

## 3 Results

From a total number of 4,463 study population, 57.1% were women with a mean age of 44.7 (SD, 13.1) years, and 42.9% were men with a mean age of 46.2 (SD, 14.3) years. A more detailed comparison of baseline characteristics between genders is shown in **Supplementary Table 1**. Accordingly, significant differences were found in all of the baseline characteristics between genders. The baseline characteristics of responders and non-responders are shown in **Supplementary Table 2**. The responders had higher BMI, higher prevalence of FH-T2DM, and lower HDL-C, but higher physical activity compared to their non-respondent counterparts. Differences in the distribution of education and marital status were also observed between respondents and non-respondents.

The baseline characteristics of women and men by changes in the MetS status are presented in **Tables 1** and **2**, respectively. In both genders, the MetS-stable group, compared to the MetS-free group, were generally older and had higher BMI, WC, SBP, DBP, FPG, TG, and lower values of HDL-C. Moreover, the former group was more educated and reported higher frequencies for consumption of anti-hypertensive and lipid-lowering medications than the latter. Also, in both men and women, we found significant differences between different groups in being low physically active; however, this value tended to be significant in women (*p* = 0.06).

**Table 1:** Baseline characteristics of women by change in MetS status, Tehran Lipid and Glucose Study.

Variables	MetS-free (*n* = 1,514)	MetS-developed (*n* = 195)	MetS-recovery (*n* = 266)	MetS-stable (*n* = 574)	*p*-value
Age, years	39.9 (11.6)	47.4 (12.4)	49.5 (11.5)	54.3 (11.1)	<0.001
BMI, kg/m^2^	26.4 (4.1)	30.4 (4.1)	30.0 (4.1)	31.8 (4.3)	<0.001
WC, cm	82.7 (10.8)	95.7 (8.2)	93.2 (10.2)	100.1 (9.2)	<0.001
SBP, mmHg	104.5 (12.4)	117.8 (15.8)	112.4 (15.7)	126.4 (19.9)	<0.001
DBP, mmHg	68.8 (8.9)	76.4 (9.8)	73.0 (9.0)	78.1 (10.4)	<0.001
FPG, mmol/L*	4.7 (0.5)	5.0 (0.6)	5.0 (0.5)	5.3 (0.8)	<0.001
TG, mmol/L*	1.2 (0.6)	2.1 (0.7)	1.5 (0.5)	2.5 (1.0)	<0.001
HDL-C, mmol/L	1.2 (0.3)	1.03 (0.2)	1.17 (0.3)	1.0 (0.2)	<0.001
Smoking					0.100
Current smoker	43 (2.8)	6 (3.1)	5 (1.9)	17 (3)	
Past smoker	23 (1.5)	2 (1.0)	4 (1.5)	6 (1)	
Never smoker	1,448 (95.7)	187 (95.9)	257 (96.6)	551 (96)	
Education					<0.001
<6 years	339 (22.4)	22 (11.3)	24 (9.0)	25 (4.4)	
6–12 years	901 (59.5)	101 (51.8)	142 (53.4)	219 (38.2)	
>12 years	274 (18.1)	72 (36.9)	100 (37.6)	330 (57.4)	
Marital status					<0.001
Single	176 (11.6)	3 (1.5)	3 (1.1)	10 (1.7)	
Married	1,234 (81.5)	166 (85.1)	231 (86.8)	461 (80.3)	
Widowed/divorced	104 (6.9)	26 (13.3)	32 (12.0)	103 (17.9)	
Physical activity level (low)	483 (31.9)	53 (27.2)	71 (26.7)	199 (34.7)	0.063
FH-T2DM (yes)	268 (17.7)	40 (20.5)	41 (15.4)	96 (16.7)	0.510
Anti-hypertensive drug use (yes)	13 (0.9)	17 (8.7)	6 (2.3)	81 (14.1)	<0.001
Lipid-lowering drug use (yes)	27 (1.8)	9 (4.6)	10 (3.8)	70 (12.2)	<0.001

MetS, metabolic syndrome; BMI, body mass index; WC, waist circumference; SBP, systolic blood pressure; DBP, diastolic blood pressure; FPG, fasting plasma glucose; TG, triglycerides; HDL-C, high-density lipoprotein cholesterol; FH-T2DM, family history of type 2 diabetes mellitus; SD, standard deviation; IQR: interquartile range.

The characteristics are presented at phase 3 (defined as index year).

Data are shown as mean (SD) for continuous variables or number (percent) for categorical variables.

*Data are shown as median (IQR) due to skewed distribution, and comparisons were done by Kruskal–Wallis test.

**Table 2:** Baseline characteristics of men by change in MetS status, Tehran Lipid and Glucose Study.

Variables	MetS-free (*n* = 861)	MetS-developed (*n* = 214)	MetS-recovery (*n* = 250)	MetS-stable (*n* = 589)	*p*-value
Age, years	43.0 (14.8)	44.8 (13.1)	49.1 (13.5)	50.1 (13.2)	<0.001
BMI, kg/m^2^	24.8 (3.9)	27.5 (3.3)	27.2 (3.4)	29.1 (3.5)	<0.001
WC, cm	89.5 (10.0)	97.6 (7.4)	96.4 (8.6)	101.7 (7.9)	<0.001
SBP, mmHg	112.4 (14.6)	119.7 (14.2)	117.3 (15.4)	125.3 (18.3)	<0.001
DBP, mmHg	72.4 (9.1)	77.6 (8.4)	75.3 (8.9)	80.0 (10.3)	<0.001
FPG, mmol/L*	4.9 (0.6)	5.1 (0.7)	5.0 (0.6)	5.2 (0.8)	<0.001
TG, mmol/L*	1.3 (0.6)	2.3 (0.9)	4.5 (0.5)	2.6 (1.3)	<0.001
HDL-C, mmol/L	1.1 (0.2)	0.9 (0.2)	1.02 (0.2)	0.9 (0.1)	<0.001
Smoking					0.100
Current smoker	182 (21.1)	53 (24.8)	50 (20.0)	121 (20.5)	
Past smoker	131 (15.2)	38 (17.8)	40 (16.0)	123 (20.9)	
Never smoker	548 (63.7)	123 (57.6)	160 (64.0)	345 (58.6)	
Education					<0.001
<6 years	254 (29.5)	49 (22.9)	57 (22.8)	129 (21.9)	
6–12 years	463 (53.8)	137 (64.0)	136 (54.4)	314 (53.3)	
>12 years	144 (16.7)	28 (13.1)	57 (22.8)	146 (24.8)	
Marital status					<0.001
Single	188 (21.8)	22 (10.3)	20 (8.0)	27 (4.6)	
Married	658 (76.5)	187 (87.4)	228 (91.2)	557 (94.6)	
Widowed/divorced	15 (1.7)	5 (2.3)	2 (0.8)	5 (0.8)	
Physical activity level (low)	322 (37.4)	103 (48.1)	93 (37.2)	245 (41.6)	0.021
FH-T2DM (yes)	139 (16.1)	35 (16.4)	50 (20)	101 (17.1)	0.549
Anti-hypertensive drug use (yes)	10 (1.2)	2 (0.9)	7 (2.8)	28 (4.8)	<0.001
Lipid-lowering drug use (yes)	3 (0.3)	8 (3.7)	4 (1.6)	26 (4.4)	<0.001

MetS, metabolic syndrome; BMI, body mass index; WC, waist circumference; SBP, systolic blood pressure; DBP, diastolic blood pressure; FPG, fasting plasma glucose; TG, triglycerides; HDL-C, high-density lipoprotein cholesterol; FH-T2DM, family history of type 2 diabetes mellitus; SD, standard deviation; IQR: interquartile range.

The characteristics are presented at phase 3 (defined as index year).

Data are shown as mean (SD) for continuous variables or number (percent) for categorical variables.

*Data are shown as median (IQR) due to skewed distribution, and comparisons were done by Kruskal–Wallis test.

Over 3 years, among women, the MetS status changed for 461 participants. Of the 840 women with MetS at phase 2, about 31.7% recovered from MetS at phase 3. In contrast, of the 1,709 women without MetS at phase 2, about 11.4% developed MetS by phase 3. Similarly, for men participants, the MetS status changed for 464 individuals. Of the 839 men with MetS at phase 2, about 29.8% recovered from MetS at phase 3. Conversely, of the 1,075 men without MetS at phase 2, about 19.9% developed MetS by phase 3 (**Figure 1**).

During a median follow-up of 9.3 (IQR, 8.3–10.2) years after the index year for the whole population, 625 T2DM events (351 women) occurred, among whom only about 25% (*n* = 156) were on glucose-lowering medication. Crude incidence rates of T2DM were 15.54 (13.96–17.26) and 16.21 (14.35–18.25) per 1,000 person-years for women and men, respectively. Moreover, as shown in **Figure 2**, in both genders, the highest incidence rates of T2DM were found in those with MetS-stable status; the corresponding values for men and women were 32.5 (27.6–38.0) and 40.3 (34.7–46.6) per 1,000 person-years, respectively. The lowest incidence rates for T2DM were found for MetS-free status; corresponding values for men and women were 5.7 (4.1–7.6) and 6.0 (4.8–7.4), respectively. Moreover, compared with the MetS-free group, the multivariate HRs of the MetS-developed, -recovery, and -stable groups for T2DM were 2.90, 2.60, and 4.92 for men; the corresponding value for women were 2.73, 2.88, and 5.21, respectively (all *p*-values < 0.01). We did not find a statistically significant gender difference in the association between changes in MetS status and the risk of T2DM.

**Table 3** presents the association of changes in MetS with the risk of T2DM. In both men and women, in multivariate analysis (model 2), those with persistent components of MetS during 3 years, excluding low HDL-C for men, had a significantly higher risk of T2DM; moreover, the HR (95% CI) for high BP-stable status among women was marginally significant [1.33 (1.00–1.76), *p* = 0.05]. Among different components of MetS, stability in high FPG in women and men had the highest risk for incident T2DM [HR: 7.71 (5.84–10.17)] and [HR: 9.42 (6.90–12.86), respectively]. Among those who recovered from MetS components, in both genders, previous history of high WC and high FPG was significantly associated with T2DM incidence; moreover, among men, previous MetS history in the high BP-recovery group was also associated with significant risk [HR: 1.76 (1.20–2.60)]. For those who developed MetS components, in women, the development of high FPG [HR: 4.57 (3.32–6.30)] and high TG [HR: 1.63 (1.14–2.32)], and for men, developed high BP [HR: 2.53 (1.80-3.55)] and high FPG [HR: 4.21 (3.01-5.88)] were associated with significant T2DM risk. In our data analysis (**Table 3**), women with developed high BP and stable high BP components had a significantly lower risk for T2DM compared to men counterparts [women-to-men RHRs: 0.43 (0.26–0.72) and 0.58 (0.39–0.86), respectively]. However, a non-significant higher risk of T2DM was found among women with stable high TG and stable low HDL-C compared to men counterparts [women-to-men RHR: 1.44 (0.98–2.14) and 1.67 (0.98–2.86), both *p*-values = 0.06].

**Table 3:** Association between changes in MetS components and incident T2DM, Tehran Lipid and Glucose Study.

	Women (*n* = 2,549)				Men (*n* = 1,914)				
MetS components	E/N	Incidence Rate Per 1,000 Person-Years	Model 1	Model 2	E/N	Incidence Rate Per 1,000 Person-Years	Model 1	Model 2	W-to-M RHR*
			HR (95% CI)	HR (95% CI)			HR (95% CI)	HR (95% CI)	
High WC									
High WC-free	70/1,123	6.8 (5.3–8.6)	Reference	Reference	23/497	5.0 (3.2–7.5)	Reference	Reference	–
High WC-developed	24/202	13.3 (8.5–19.9)	**2.59 (1.46–4.59)**	1.49 (0.94–2.38)	16/202	8.4 (5.0–14.2)	1.86 (0.98–3.51)	1.45 (0.76–2.76)	1.02 (0.46–2.27)
High WC-recovery	26/204	14.0 (9.1–20.5)	**2.75 (1.57–4.82)**	1.58 (1.00–2.48)†	10/47	24.6 (11.8–45.3)	**4.17 (1.98–8.77)**	**2.85 (1.35–6.03)**	0.55 (0.23–1.32)
High WC-stable	231/1,020	26.8 (23.5–30.5)	**4.47 (2.91–6.87)**	**1.85 (1.38–2.48)**	225/1,168	22.3 (19.5–25.4)	**4.02 (2.61–6.17)**	**2.67 (1.73–4.13)**	0.69 (0.41–1.15)
High BP									
High BP-free	186/1,804	11.5 (9.9–13.2)	Reference	Reference	99/1,196	9.1 (7.4–11.1)	Reference	Reference	
High BP-developed	32/172	21.7 (14.8–30.6)	**2.02 (1.35–3.02)**	1.11 (0.75–1.63)	53/210	30.8 (23.1–40.3)	**2.80 (2.00–3.93)**	**2.52 (1.80–3.54)**	**0.43 (0.26–0.72)**
High BP-recovery	41/243	19.7 (13.7–25.9)	**1.75 (1.21–2.53)**	1.15 (0.81–1.62)	35/182	22.8 (15.9–31.8)	**2.20 (1.49–3.24)**	**1.76 (1.20–2.60)**	0.64 (0.38–1.08)
High BP-stable	92/330	33.8 (27.2–41.4)	**2.62 (1.94–3.54)**	1.33 (1.00–1.76)†	87/326	31.6 (25.3–38.9)	**2.55 (1.88–3.44)**	**2.26 (1.66–3.08)**	**0.58 (0.39–0.86)**
High FPG									
High FPG-free	178/2,156	9.0 (7.7–10.4)	Reference	Reference	126/1,530	9.0 (7.5–10.7)	Reference	Reference	–
High FPG-developed	50/120	57.9 (43.0–76.3)	**5.74 (4.13–7.98)**	**4.56 (3.30–6.28)**	49/137	45.3 (33.5–59.8)	**4.61 (3.31–6.43)**	**4.24 (3.04–5.93)**	1.07 (0.67–1.70)
High FPG-recovery	41/143	33.6 (24.1–45.7)	**3.36 (2.36–4.79)**	**2.49 (1.76–3.53)**	36/144	29.3 (20.6–40.6)	**2.90 (2.00–4.20)**	**2.66 (1.83–3.87)**	0.93 (0.56–1.55)
High FPG-stable	82/130	110.3 (87.7–136.9)	**10.407 (7.88–13.92)**	**7.72 (5.85–10.19)**	63/103	102.1 (78.4–130.6)	**10.24 (7.54–13.92)**	**9.51 (6.96–12.99)**	0.81 (0.53–1.22)
High TG									
High TG-free	91/1,352	7.4 (5.9–9.0)	Reference	Reference	80/820	10.8 (8.6–13.5)	Reference	Reference	–
High TG-developed	49/296	18.8 (13.9–24.9)	**1.73 (1.21–2.47)**	**1.63 (1.14–2.32)**	35/220	18.5 (12.9–25.7)	**1.83 (1.23–2.72)**	1.34 (0.89–2.02)	1.21 (0.70–2.07)
High TG-recovery	38/264	16.2 (11.5–22.3)	1.45 (0.99–2.13)††	1.34 (0.91–1.97)	39/237	18.8 (13.3–25.7)	**1.72 (1.18–2.53)**	1.37 (0.93–2.02)	0.97 (0.56–1.68)
High TG-stable	173/637	32.7 (28.0–38.0)	**2.67 (2.05–3.49)**	**2.12 (1.61–2.79)**	120/637	21.6 (17.9–25.9)	**1.99 (1.50–2.64)**	**1.47 (1.09–1.97)**	1.44 (0.98–2.14)††
Low HDL-C									
Low HDL-C-free	24/339	7.9 (5.1–11.8)	Reference	Reference	43/354	13.9 (10.1–18.7)	Reference	Reference	–
Low HDL-C-developed	19/164	13.0 (7.8–20.2)	1.04 (0.61–1.79)	1.16 (0.63–2.12)	15/132	12.7 (7.1–21.0)	1.09 (0.61–1.97)	0.85 (0.47–1.53)	1.36 (0.58–3.18)
Low HDL-C-recovery	40/423	10.5 (7.5–14.4)	0.87 (0.57–1.35)	1.15 (0.69–1.90)	47/349	15.1 (11.1–20.0)	1.13 (0.75–1.71)	1.01 (0.66–1.53)	1.13 (0.59–2.19)
Low HDL-C-stable	268/1,623	18.7 (16.6–21.1)	**1.61 (1.16–2.23)**	**1.68 (1.10–2.57)**	169/1,079	17.8 (15.2–20.6)	**1.46 (1.04–2.04)**	1.00 (0.71–1.42)	1.67 (0.97–2.86)††

MetS, metabolic syndrome; T2DM, type 2 diabetes mellitus; E, event; N, total number of population; HR, hazard ratio; CI, confidence interval; W-to-M RHR, women-to-men ratio of hazard ratios; WC, waist circumference; BP, blood pressure; FPG, fasting plasma glucose; TG, triglycerides; HDL-C, high-density lipoprotein cholesterol.

Model 1: Adjusted for age.

Model 2: Adjusted for age, smoking status, physical activity level, education, marital status, family history of T2DM, body mass index, being in the intervention group + other components of MetS.

*Women-to-men RHR: The value shows women-to-men relative HR for each parameter obtained in model 2.

† p-value = 0.05, †† p-value = 0.06.

Bold values are statistically significant (P ≤ 0.05).

## 4 Discussion

In the current study conducted in a population-based cohort in a population with a high burden of MetS, we examined the association between changes in MetS status (JIS criteria) and their components over approximately 3 years with incident T2DM during near one decade of follow-up. Moreover, we examined the potential effect modification of gender in the mentioned relationship. Accordingly, we found that in both genders compared to MetS-free, other groups were significantly associated with a greater risk of incident T2DM up to fivefold among the MetS-stable group, with no existing gender difference in this relationship. Regarding the MetS components, in both genders, compared with the reference, for the FPG component, all of the other groups and for the WC component, those with high WC-recovered and -stable were significantly associated with incident T2DM. For BP component among men, all the other groups and for women, only the high BP-stable group and considering lipid components, among men only high TG-stable and for women, both high TG-stable and -developed groups as well as low HDL-C-stable groups were significant predictors. Generally, the impact of high BP components among men and dyslipidemia components among women was more prominent in the development of T2DM, although the latter was marginally significant.

Prior works have documented the strong predictive ability of MetS for T2DM (3, 6). In this regard, it has even been suggested to consider T2DM as a major outcome of MetS rather than a component to maximize the clinical effectiveness of this ability (28). However, few studies have investigated the effect of changes in the status of MetS during a period on subsequent T2DM incidence in the follow-up (17–20).

Based on 2-year changes in MetS status [National Cholesterol Education Program Adult Treatment Panel III (NCEP-ATP III) criteria], Huh et al. divided their Korean study population of 7,317 adults into four groups: non-MetS, resolved MetS, incident MetS, and persistent MetS. Compared to the non-MetS group, all other categories had increased T2DM risk in a pooled analysis. Ohnishi et al. had similar findings in a Japanese cohort. However, they found that having central adiposity as a core component of MetS as suggested by International Diabetes Federation (IDF) criteria rather than being one of the components was associated with a higher impact of MetS on the development of diabetes (19). It is of note that a previous MetS history among those recovered from MetS in both mentioned studies was associated with a non-significant increased T2DM risk; however, in our study, prior history of MetS was still associated with more than a twofold risk of T2DM. Lee et al., among more than 10 million of the South Korean population, examined the effect of changes in MetS and its components between two visits during 2 years with the subsequent risk of T2DM during an average follow-up of 4 years. Compared to MetS-stable or those who persistently had the MetS components during the 4 years, all other transition statuses were significantly associated with a lower risk of T2DM. The greatest risk reduction among those recovered from MetS or its components occurred in those with improvement in high FPG, which significantly reduced the T2DM risk by 46% (18). Similarly, in another study conducted among the Chinese population, the researchers found that dynamic changes of FPG had the highest predictive ability for detecting T2DM, followed by the dynamic change of MetS (20). We extend the previous studies by showing that in Iranian men and women, compared to MetS-free, all other groups, even the MetS-recovered, are at three- to fivefold increased risk for incident T2DM. Moreover, as expected among different components of MetS, generally, the FPG component, regardless of status change, had the strongest association with incident T2DM, with the HRs reaching 7 and 9 in men and women, respectively.

The association between individual components of MetS and incident T2DM was reported in many studies (21, 29). In our data analysis, recovery from high WC and high FPG in both genders, in addition to recovery from high BP in men, still conferred significant risks for T2DM. “Metabolic memory” might be a plausible explanation for the continued increased risk even after recovery from impaired metabolic/glycemic control (30–33).

We found that the development and stability of high BP had greater association with incident T2DM in men than in women (women-to-men RHRs: 0.43 and 0.58, respectively). Similarly, the MONICA study suggested that baseline SBP significantly predicted diabetes in men but not in women (34). Also, Bogalusa Heart Study reported that greater SBP in childhood resulted in a higher risk of T2DM in men than in women later in adulthood (35). Therefore, high BP, especially among men, might signal an increased risk for T2DM. However, conflicting results have been reported regarding the gender differential adverse impact of BP on the risk of T2DM, with reports of SBP being a stronger driver for T2DM in women (36) or not different between genders (37).

In our data analysis compared to men, women with high TG-stable and low HDL-C-stable had about 40% and 70% higher risks for the development of T2DM, respectively. Reduced HDL-C and elevated TG levels are common dyslipidemic features in T2DM (38), and the coexistence of both conditions has been described as a surrogate of IR (39). In those without T2DM, women have been reported to have generally higher HDL-C and lower TG levels than men (40, 41). However, women’s metabolic risk profile before progression to T2DM has been described to be worse than men’s (42–47). For example, in the Maastricht study, women progressing to diabetes experienced greater adverse changes in HDL-C and TG than men (43). Dissimilarities in sex differences in the relation between HDL-C and T2DM were also found in studies, with some reporting more beneficial effects of greater HDL-C levels on reducing T2DM risk in female individuals (34, 36), while others found the mentioned protective effects only in male individuals (37) or no difference between sexes (35).

Disparities witnessed in our study might be, in some extent, due to the already explored gender-specific differences in cardiometabolic health (48, 49), including sex hormones (50), body composition (51), lipid metabolism (48), adipose tissue distribution (51), and energy expenditure (52).

The current study has some strengths; the main one being its prospective population-based cohort design with a large sample size and a relatively long follow-up period. Additionally, the standardized measurements, including demographic and anthropometric measurements, were gathered using standard questionnaires and laboratory assays rather than relying on self-reported data. Finally, to our knowledge, this study is the first to pursue the gender differences in the relationship between status changes of MetS and its components and incident T2DM. There are also limitations in the current study that should be acknowledged. Firstly, since the study population was from the metropolitan city of Tehran, the generalization of the findings may not necessarily apply to the rural zones of the country, or other ethnic groups. Secondly, about 40% of our eligible participants did not enter our data analysis; however, we did not find significant differences in major risk factors of T2DM between respondents versus non-respondents in terms of age, WC, FPG, SBP, and DBP. Thirdly, the data of other important residual confounders, such as dietary factors and psycho-socio-economic status, were unavailable.

## 5 Conclusions

In our study, men and women with a history of MetS, including MetS-stable, MetS-developed, and even recovered groups, are at increased risk for T2DM. In both genders, statuses of developed, recovered, and stable high FPG, in addition to recovered and stable high WC, are strongly associated with risk of incident T2DM. Regarding gender differences in MetS components, stability and development of high BP among men and the stable status of dyslipidemia among women have a greater risk for T2DM. Therefore, additional studies are necessary to confirm gender differences in MetS components for the development of T2DM and clarify the possible explanations for mechanisms behind the differential effects.

## Data availability statement

The raw data supporting the conclusions of this article will be made available by the authors, without undue reservation.

## Ethics statement

The studies involving human participants were reviewed and approved by The Institutional Review Board of the Research Institute for Endocrine Sciences (RIES), Shahid Beheshti University of Medical Sciences, Tehran, Iran. The patients/participants provided their written informed consent to participate in this study.

## Author contributions

FH, MT, and FA participated in the conception and design of the study; AA, PH, and MT carried out the literature search; AA, PH, and KK participated in data collection; KK carried out the data analysis; FH, MT, AA, PH, and KK participated in interpretation of the analyses and writing the manuscript; FH, MT, AA, PH, KK, and FA participated in final approval of the version to be submitted. All authors read and approved the final manuscript.
