# Supplementary material for: Gender differences in the impact of 3-year status changes of metabolic syndrome and its components on incident type 2 diabetes mellitus: a decade of follow-up in the Tehran Lipid and Glucose Study
Source: Front Endocrinol (Lausanne). 2023 May 25;14:1164771. doi: 10.3389/fendo.2023.1164771 (PMC10248400; doi:10.3389/fendo.2023.1164771)
Supplement: Supplementary file 1 [file Table_1.docx]

Supplementary Material

Gender differences in the impact of three-year status changes of metabolic syndrome and its components on incident type 2 diabetes mellitus: A decade of follow-up in the Tehran Lipid and Glucose Study

**Farzad Hadaegh^1†^, Amir Abdi^1†^, Karim Kohansal^1^, Parto Hadaegh^1^, Fereidoun Azizi^2^, Maryam Tohidi^1^***

*** Correspondence:** Maryam Tohidi: [tohidi@endocrine.ac.ir](mailto:tohidi@endocrine.ac.ir)

| Supplementary Table 1. Baseline characteristics of study population by gender, Tehran Lipid and Glucose Study | | | |
| --- | --- | --- | --- |
| Variables | **Women (n=2549)** | **Me**n **(n=1914)** | **P-value** |
| Age, year | 44.7 (13.1) | 46.2 (14.4) | <0.001 |
| BMI, kg/m^2^ | 28.3 (4.8) | 26.8 (4.1) | <0.001 |
| WC, cm | 88.7 (12.7) | 95.1 (10.4) | <0.001 |
| SBP, mmHg | 111.3 (17.5) | 117.8 (16.8) | <0.001 |
| DBP, mmHg | 71.9 (10.1) | 75.7 (9.9) | <0.001 |
| FPG, mmol/L* | 4.9 (0.6) | 5.0 (0.6) | <0.001 |
| TG, mmol/L* | 1.6 (1.0) | 1.9 (1.1) | <0.001 |
| HDL-C, mmol/L | 1.16 (0.27) | 0.98 (0.22) | <0.001 |
| Smoking |  |  | <0.001 |
| Current smoker | 71 (2.8) | 406 (21.2) |  |
| Past smoker | 35 (1.4) | 332 (17.3) |  |
| Never smoker | 2443 (95.8) | 1176 (61.4) |  |
| Education |  |  | <0.001 |
| < 6 years | 410 (16.1) | 489 (25.5) |  |
| 6-12 years | 1363 (53.5) | 1050 (54.9) |  |
| > 12 years | 776 (30.4) | 375 (19.6) |  |
| Marital status |  |  | <0.001 |
| Single | 192 (7.5) | 257 (13.4) |  |
| Married | 2092 (82.1) | 1630 (85.2) |  |
| Widowed/divorced | 265 (10.4) | 27 (1.4) |  |
| Physical activity level (low) | 806 (31.6) | 763 (39.9) | <0.001 |
| FH-T2DM (yes) | 445 (17.5) | 325 (17.0) | 0.705 |
| Anti-hypertensive drug use (yes) | 117 (4.6) | 47 (2.5) | <0.001 |
| Lipid-lowering drug use (yes) | 116 (4.6) | 41 (2.1) | <0.001 |
| MetS, metabolic syndrome; BMI, body mass index; WC, waist circumference; SBP, systolic blood pressure; DBP, diastolic blood pressure; FPG, fasting plasma glucose; TG, triglycerides; HDL-C, high-density lipoprotein cholesterol; FH-T2DM, family history of type 2 diabetes mellitus; SD, standard deviation; IQR: interquartile range.  The characteristics are presented at phase 3 (defined as index year).  Data are shown as mean (SD) for continuous variables or number (percent) for categorical variables.  * Data are shown as median (IQR) due to skewed distribution. | | | |

| Supplementary Table 2. Baseline characteristics of respondents and non-respondents: Tehran Lipid and Glucose Study | | | |
| --- | --- | --- | --- |
| Variables | **Respondents (n=4463)** | **Non-respondents (n=3349)** | **P-value** |
| Continuous variables |  |  |  |
| Age, year | 45.3 (13.6) | 46.0 (16.5) | 0.147 |
| BMI, kg/m^2^ | 27.6 (4.6) | 27.3 (5.0) | 0.045 |
| WC, cm | 91.4 (12.2) | 90.8 (13.2) | 0.112 |
| SBP, mmHg | 114.1 (17.5) | 114.8 (19.5) | 0.244 |
| DBP, mmHg | 73.6 (10.2) | 73.2 (10.9) | 0.299 |
| FPG, mmol/L* | 4.9 (0.5) | 4.9 (0.5) | 0.977 |
| TG, mmol/L* | 1.7 (1.0) | 1.7 (1.1) | 0.294 |
| HDL-C, mmol/L | 1.08 (0.26) | 1.10 (0.27) | 0.019 |
| Categorical variables |  |  |  |
| Smoking |  |  | 0.166 |
| Current smoker | 477 (10.7) | 178 (12.4) |  |
| Past smoker | 367 (8.2) | 109 (7.6) |  |
| Never smoker | 3619 (81.1) | 1147 (80.0) |  |
| Education |  |  | 0.033 |
| < 6 years | 899 (20.1) | 282 (19.1) |  |
| 6-12 years | 2413 (54.1) | 763 (51.7) |  |
| > 12 years | 1151 (58.8) | 432 (29.2) |  |
| Marital status |  |  | <0.001 |
| Single | 449 (10.1) | 209 (13.2) |  |
| Married | 3722 (83.4) | 1227 (77.8) |  |
| widowed/divorced | 292 (6.5) | 141 (9.0) |  |
| Physical activity level (low) | 1569 (35.2) | 518 (38.7) | 0.019 |
| FH-T2DM (yes) | 770 (17.3) | 238 (15.1) | 0.049 |
| Anti-hypertensive drug use (yes) | 164 (3.7) | 75 (4.7) | 0.071 |
| Lipid-lowering drug use (yes) | 157 (3.5) | 59 (3.7) | 0.749 |
| BMI, body mass index, WC, waist circumference; SBP, systolic blood pressure; DBP, diastolic blood pressure; FPG: fasting plasma glucose; TG, triglycerides; HDL-C, high-density lipoprotein cholesterol; FH-T2DM, family history of T2DM; SD, standard deviation; IQR, interquartile range.  The baseline characteristics are presented at phase 3 (defined as index year).  Data are shown as mean (SD) for continuous variables or number (percent) for categorical variables.  * Data are shown as median (IQR) due to skewed distribution and comparisons were done by Mann-Whitney U test. | | | |
